# Experience With Store-and-Forward Consultations in Providing Access to Pediatric Endocrine Consultations in Low- and Middle-Income Countries

**DOI:** 10.3389/fpubh.2019.00272

**Published:** 2019-09-25

**Authors:** Julia E. von Oettingen, Meghan Craven, Regina Duperval, Florence Sine St. Surin, Ronald Eveillard, Rony Saint Fleur, Guy Van Vliet, Jean-Pierre Chanoine, Renault Louis

**Affiliations:** ^1^McGill University Health Centre Research Institute, Montreal, QC, Canada; ^2^Division of Global Health, Boston Children's Hospital, Boston, MA, United States; ^3^Department of Pediatrics, Hôpital St. Damien, Port-au-Prince, Haiti; ^4^Department of Pediatrics, Hôpital Universitaire La Paix, Port-au-Prince, Haiti; ^5^Department of Pediatrics, Hôpital de l'Université d'État d'Haiti, Port-au-Prince, Haiti; ^6^Department of Pediatrics, Hôpital Universitaire Justinien, Cap-Haitien, Haiti; ^7^Division of Endocrinology, Centre Hospitalier Universitaire Sainte Justine, Montreal, QC, Canada; ^8^Department of Pediatrics, British Columbia Children's Hospital, Vancouver, BC, Canada; ^9^Department of Pediatrics, Mirebalais University Hospital, Mirebalais, Haiti

**Keywords:** pediatric endocrinology, childhood diabetes, low-resource setting, teleconsultation, store-and-forward networks

## Abstract

Pediatric specialists are often unavailable in low- and middle-income countries. As part of multiple professional associations' efforts to improve access to endocrine expertise globally, a pediatric endocrine teleconsultation network was established on a store-and-forward teleconsultation platform to facilitate focused, language-appropriate advice that can be kept for future reference while bypassing real-time video-conferencing, and obviating the need for a scheduled appointment. User information was recorded, and quality statistics on network performance and qualitative evaluation by referring physicians were analyzed. Over a 3-year period, 81 referrers (88% from Haiti) and 13 pediatric endocrinologists registered onto the network and discussed 47 pediatric endocrine cases, exchanging a total of 412 messages for a median of 7 messages (IQR 5, 11) per case. Diagnoses spanned the spectrum of pediatric endocrine disorders. According to referrers, an appropriate expert was consulted and an answer provided sufficiently quickly in 100% of cases. The answer was well-adapted to their environment in 86%, and referrers were able to follow the advice given in 72%. All but one referrer found the advice helpful, it clarified the diagnosis in 88%, assisted with management in 93%, improved patient's symptoms in 77%, improved function in 77%, and was considered cost-saving in 50%. Perceived benefits of the consultations were academic instruction, setting-adapted advice beyond the scope of guidelines or textbooks, and advancement in the diagnostic process. Pediatric endocrine remote store-and-forward consultations in low- and middle-income countries may provide a reasonable alternative to face-to-face visits, providing clinical and educational benefit, and a potential for cost-saving.

## Introduction

In most low- and middle-income countries, access to pediatric subspecialty care is severely limited or entirely unavailable. Only a small number of trained pediatric subspecialists, if any, may be residing in-country. Even the most basic subspecialty education for health professionals including nurses, medical students, physicians in training, and in practice may be lacking when no teacher has been available for extended periods of time. As a result, medical conditions which are beyond the scope of most medical practitioners frequently go unrecognized or are mis-diagnosed, leading to potentially preventable morbidity, and mortality.

Telemedicine consultations can offer access access to specialty consultation in high-income countries (1, 2), and in low- and middle-income countries can fill a significant access gap, enabling physicians to care for patients when local subspecialty expertise is unavailable (3). However, reports of the successful use of teleconsultation services for pediatric endocrinology are limited (4, 5) and reports from low- and middle-income countries are not available.

**Figure 1:**
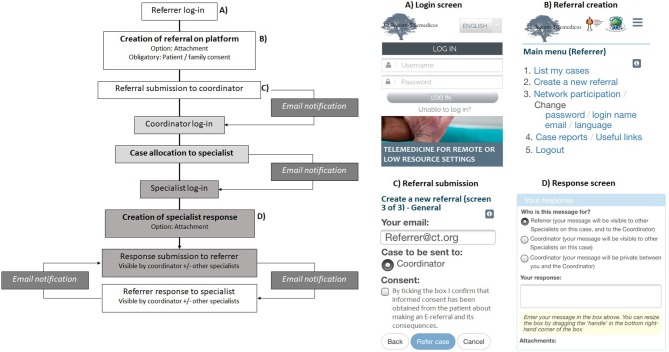
Schematic diagram of store-and-forward teleconsultation process. The referral process from creation of a new referral to specialist response is shown. Screen shots of the platform as viewed by users on a mobile phone are shown in **(A–D)**.

Collegium Telemedicus (www.collegiumtelemedicus.org) is a store-and-forward consultation platform that is available on the web and on mobile devices, offering the opportunity to confidentially discuss clinical cases in a secure manner (6) while avoiding the downsides of informal, undocumented “curbside” consultations. As opposed to real-time consultation, “store-and-forward” consultations are asynchronous and imply that the referring physician creates a consultation request that is *stored* on the platform until *forwarded* to a consultant (Figure 1). This approach allows the practitioner to communicate without the need for a scheduled appointment, circumvents the problem of low-speed internet connection not supporting real-time video-conferencing, allows the practitioner to return to the consultation document as and when required, and facilitates follow-up communication with the specialist as needed for patients requiring ongoing care.

In Haiti, a low-income country with a population of 11 million in Central America where one third of the population is under 15 years old, no pediatric endocrinologist is available. Endocrine disorders such as type 1 diabetes, hypoglycemia and adrenal disorders—all manageable conditions when adequately taken care of—are frequently fatal. Other conditions such as thyroid disease or disorders of sexual development are left untreated, causing significant morbidity. In 2015, the Pediatric Endocrine Society's International Relations Council (PES IRC) began working with Haitian medical schools, pediatric residency programs, local professional associations, the non-governmental organization Zanmi Lasante (Partners in Health), and the Ministry of Public and Population Health to develop the Pediatric Endocrinology Education Program for Haiti (PEEP-H, www.peephaiti.org). In partnership with the European Society for Pediatric Endocrinology (ESPE), a comprehensive training program was developed to establish pediatric endocrinology as a specialty in Haiti (7). The 4 year program consists of an onsite and remote training curriculum provided by a body of francophone pediatric endocrinologists to Haitian health professionals at all levels of training.

From the time of program initiation in 2016, PEEP-H has collaborated with CollegiumTelemedicus to use its platform to offer remote clinical consultation services and support in the diagnosis and management of pediatric patients affected by endocrine conditions in Haiti, while providing case-based teaching and education to the referring physicians. By 2017, access to the PEEP-H telemedicine network was opened up to any referring physician from a low- or middle-income country via a collaboration with the non-governmental organization Global Pediatric Endocrinology and Diabetes (GPED, www.globalpedendo.org).

The objective of this study was to critically assess the use of a pediatric endocrinology telemedicine network and evaluate its impact on clinical care and pediatric endocrine education in Haiti and beyond.

## Materials and Methods

### Ethical Considerations

Given the quality improvement nature of the study, ethics review was waived by the Montreal Children's Hospital ethics review board. Referring physicians obtained verbal consent from all families to discuss their child's case with specialist consultants through teleconsultation within the PEEP-H/GPED network.

### Description of the Store-And-Forward Teleconsultation Process

The Collegium Telemedicus platform was used to create a network space for PEEP-H and GPED for the purpose of pediatric endocrine consultation services. Invited referring and specialist physicians were asked to fill in a registration form and their accounts were then authorized by the network coordinator (JO). During this process, users are able to choose their language of communication (English, French, Spanish, or Arabic). Figure 1 provides an overview of the teleconsultation process: Upon successful login to the platform, referrers first specify the type of consultation (clinical advice for a specific case vs. concerning a group of patients), and the reason for consultation (advice on differential diagnosis, current management, need for transfer to a different level of care, general information on the pathology discussed, or other). They then use a pre-defined generic template (Table 1) to create new consult requests. Pictures, video files and documents can be uploaded as attachments as needed. Once their consult form is submitted, an email is automatically generated to alert the coordinator to a new case. The coordinator can then review the consult form and assign it to an appropriate specialist consultant. This allocation generates an email to the consultant prompting them to view and respond to the case in their account. Following this, referring physicians and consultants can communicate via messages on the platform, each message automatically generating an email alerting the receiving physician to a new message. Images, video clips, documents, and teaching materials can be attached to messages.

**Table 1:** Consult template.

1. Presenting Complaint	
2. History of Presenting Complaint	
3. Past medical history	Birth history: Gestational age __________(weeks) Birth weight ______________(gm) Birth length (if available)_____(cm) Complications during pregnancy/delivery /perinatal period (specify): Infancy: Weight gain: average/slow/fast Growth in length: average/slow/fast Development: average/slow/fast Childhood: Weight gain: average/slow/fast Growth in height: average/slow/fast Development: average/slow/fast
2. Family history	Diabetes Other endocrine disorder Autoimmune disorder Mother's height (if consulting about growth problem, measure parent) Father's height (if consulting about growth problem, measure parent)
3. Anthropometrics and vital signs	Age:(___years, ___months) Weight (kg) __ Length or height (cm) __
4. Physical examination	General appearance, including signs of dysmorphism: Head/Ears/Eyes/Nose/Throat, including thyroid: Cardiovascular and Respiratory: Abdomen: Genitourinary, including Tanner staging of breast, pubic hair, testicular volume (as indicated) Skin: Neuro:
5. Investigations	
6. Working diagnosis	
7. Current management/treatment	
8. Other	

### Data Collection

Demographic data on participants who sign on to the Collegium Telemedicus system and quality and performance measures are collected as part of the network use (8), are securely stored on the online platform and can be viewed by the network coordinator. For the purpose of this study, we collected Collegium Telemedicus-generated data on project participants, case statistics, and consultation evaluation by the referring physicians.

#### Participants

Participants included in the study were referring physicians (pediatricians, generalists, resident physicians) from low- and middle-income countries and licensed pediatric endocrinologists from any country who registered for the PEEP-H/GPED network on the Collegium Telemedicus platform between 03/2016 and 03/2019. Platform specialists were recruited among PEEP-H teaching professors, and by contacting pediatric endocrinologists among GPED members. The platform was advertised to referring users in several ways. In Haiti, PEEP-H program participants were encouraged to register during onsite and remote teaching sessions, and platform information was disseminated to general pediatricians and pediatric residents via email and at conferences offered by the Haitian Medical Association and the Haitian Pediatric Society. GPED has promoted the platform in its newsletters, on its website, and at international pediatric endocrine conferences. Lastly, in 2018 a link to the platform was created on the ESPE e-learning site that offers interactive chapters and case discussions in pediatric endocrinology to users worldwide (www.espe-elearning.org). Registration and platform account activation was overseen by the PEEP-H coordinator. Information was collected on participant's country and city of professional practice, institutional affiliation, level of training, languages comfortable to communicate in for the purpose of medical consultation, and number of cases referred or responded to.

#### Case Statistics

For each consult case received through the PEEP-H/GPED network, we collected information on the age and sex of the child, presumed diagnosis, and whether the diagnosis was confirmed. We also obtained information on the country of origin of both referring physician and consultant, on the language the consultation was written in, the number of messages exchanged, and the duration of communication between the referring physician—consultant dyad as defined by the time between the consultation was received and the last message exchanged. We registered each case as coming from a PEEP-H vs. a GPED participant.

#### Consultation Evaluation

We recorded monthly statistics on the number of cases received and on time delay (in hours) to allocation, time to consultant response, and referring physician wait time to answer. Allocation delay was defined as the time in hours from reception of a consult from a referring physician to assignment to a pediatric endocrinologist. This reflected the coordinator's performance. Time to consultant response was the time from allocation of the consult to first response by the consultant and reflected the consultant's performance. The total wait time to answer was defined as the total time from referring physicians consult request to consultant response to the referring physician and reflected both coordinator and consultant physician performance.

Three weeks after the last message was exchanged between the referring physician and the consultant, an automated evaluation questionnaire containing both quantitative and qualitative questions was sent to each referring physician (Table 2). The questionnaire targets domains of sufficiently quick response, well-adapted answer, helpful advice and ability to follow advice, and assesses whether it benefited patient outcome, whether costs for patient and/or the hospital were saved, and whether there was an educational benefit.

**Table 2:** CollegiumTelemedicus automatic evaluation questionnaire.

1	Was the case sent to an appropriate expert?
2	Was the answer provided sufficiently quickly?
3	Was the answer well-adapted for your local environment?
4	Were you able to follow the advice given?
5	If NO, could you explain briefly why no
6	Did you find the advice helpful?
7a	If YES, did it clarify your diagnosis
7b	If YES, did it assist with your management of the patient?
7c	If YES, did it improve the patient's symptoms
7d	If YES, did it improve function
7e	Any other reason? Please specify
8	Do you think the eventual outcome for the patient will be beneficial for the patient?
9	Was there any educational benefit to you in the reply?
10	Was there any cost-saving as a result of this consultation?
10a	Was there any cost saving for the patient/family
10aa	If YES, please explain briefly
10b	Was there any cost saving for the hospital/clinic
10bb	If YES, please explain briefly
11	Please add any other comments about this case specifically
12	Please add any other comments about the service generally

### Urgent Advice

Although Collegium Telemedicus offers new consultation alerts via text message, a synchronous text message exchange between users is not currently possible. However, we anticipated that urgent questions for cases such as diabetic ketoacidosis, hypoglycemia, and adrenal crisis would require fast response times and a direct exchange via text message or phone call. We thus established a less formal platform for urgent questions using an independent encrypted text messaging group on WhatsApp in August 2017. Concurrent with their Collegium Telemedicus registration, participants could provide their phone number to be added to the text messaging group. Participants were reminded to send urgent questions without patient identifiers or images, and were encouraged to subsequently submit their full consult via the Collegium Telemedicus platform. One pediatric endocrinologist per week was assigned as coordinator of the text messaging group.

### Statistical Analysis

We used standard descriptive statistics for participant demographic information. We calculated means and standard deviations, median and interquartile ranges, and percentages, as appropriate, for case statistics and the quantitative data of the consultation evaluation. We structured the qualitative evaluation data by reviewing referring physician comments and assigning codes to broad concepts that appeared in the answers. We then identified themes to describe the most common responses to questions, patterns of codes in the answers provided, and areas that merit further exploration.

## Results

### Participants

A total of 81 referrers and 18 pediatric endocrinologists were signed on to the PEEP-H/GPED network, of whom 71 (88%) and 13 (72%), respectively, were part of the PEEP-H network and the remainder were part of the GPED network from countries including Myanmar, Pakistan, Algeria, Egypt, Liberia, Mexico, Greece, Syria, and India (Table 3). The majority of referring physicians (*n* = 72, 88%) were from Haiti, of whom 31 (38%) were resident physicians and 49 (60%) were pediatricians and the most commonly used language of communication was French. Consultant pediatric endocrinologists were mostly from North America, and 16 (89%) were practicing at a University Hospital.

**Table 3:** Participant characteristics.

	**Referring physician** **(*n* = 81)**	**Consultant** **(*n* = 18)**
**PHYSICIAN TYPE**		
Pediatric endocrinologist	1	18
Pediatrician	49	0
Other practicing physician	0	0
Pediatrician in training (resident)	31	0
**INSTITUTIONAL AFFILIATION**		
University Hospital	70	16
Non-university hospital	3	1
Private practice	6	1
Other	2	0
**COUNTRY OF ORIGIN**		
Haiti/Central America	72	0
North America (USA or Canada)	0	13
Europe	2	1
Australia	0	2
South-East Asia	5	0
Africa	2	2
**PRIMARY NETWORK**		
PEEP-H	70	13
GPED	11	5
**LANGUAGE**		
French	68	16
English	13	11
Spanish	0	3
Arabic	0	1
**NUMBER OF LANGUAGES**		
1	74	4
2 or more	7	14

### Cases Statistics

Over a period of 3 years, 47 cases or on average 1.2 cases per month were referred. The rate of referrals has remained constant over time. Thirty-six cases (77%) contained an attachment including patient photographs, results of laboratory investigations or imaging. The majority of cases (38 or 81%) were received from Haiti through the PEEP-H network and were discussed in French (37 or 78%). The remainder 9 cases came from Myanmar (*n* = 6), Greece (*n* = 1), and Syria (*n* = 1) through the GPED network, and they were all discussed in English. Of the referrers who sent consult requests, each sent a median of 1 (IQR 1, 2) case, and consultants responded on average to a median of 3 (IQR 1, 7) cases. The median duration of communication between referring physician and consultant was 20.9 (IQR 20.2, 24.0) days, and a total of 412 messages were exchanged, for a median of 7 messages (IQR 5, 11) per case. The median allocation delay for each case was 13.6 h (IQR 2.5, 37.5), the median time to first response was 11.3 h (IQR 2.1, 40.0), for a median total answer delay of 43.1 h (IQR 15.1, 82.8).

Patient characteristics with reasons for consultation and presumed diagnoses are shown in Table 4. The median age of referred children was 2.0 (IQR 0.2, 7.0, range 0–16) years. Twenty-three (49%) were boys, 18 (38%) girls, 5 (11%) had ambiguous genitalia, and in 1 (2%) the sex was unknown.

**Table 4:** Provisional diagnoses of referred patients by category of suspected endocrine disorder.

**Category of suspected endocrine disorder**	**Provisional diagnosis/Clinical impression**	**Age**	**Sex**
Adrenal	Simple virilizing congenital adrenal hyperplasia in 46 XX child raised as boy	7 years	M
	Salt-wasting congenital adrenal hyperplasia	5 days	Ambiguous
	Salt-wasting congenital adrenal hyperplasia	15 days	F
	Premature adrenarche, rule-out congenital adrenal hyperplasia	5 years	F
	Adrenal insufficiency	3 months	M
	Adrenal suppression	1 year 5 months	F
	Cushing's disease	15 years	M
	Cushing's syndrome due to adrenal carcinoma	2 years 1 month	M
	Pseudo-hypoaldosteronism	1 month	F
Bone	Rickets, nutritional	3 years	M
	Osteogenesis imperfecta, type I	20 days	F
	Osteogenesis imperfecta, type IV	9 years	M
Cardiovascular disease	Hypertension	12 years	M
Diabetes	Type 1 diabetes	7 years	F
	Neonatal hyperglycemia due to infection	2 months	M
	New onset type 1 diabetes	4 years	F
	Type 1 diabetes	1 year, 3 months	F
	Neonatal diabetes	1 month	M
	Neonatal diabetes	2 months	M
Disorders of sexual development	Ambiguous genitalia	17 days	F
	Ambiguous genitalia, under-virilized male	n/a	Ambiguous
	Ambiguous genitalia, under-virilized male	5 days	Ambiguous
	Ambiguous genitalia, VACTERL (Vertebral defects, Anal atresia, Cardiac defects, Tracheo-esophageal fistula, Renal anomalies, and Limb abnormalities) likely	20 days	Ambiguous
	Under-virilized male	16 years	M
	Vestigial tail (9)	n/a	F
Growth	Growth retardation, possible hypopituitarism	12 years	M
Hypoglycemia	Hyperinsulinemic hypoglycemia	2 years	M
	Hyperinsulinism,? Beckwith-Wiedemann	1 days	F
Lipids	Familial hypercholesterolemia	8 years	F
	Mucopolysaccharidosis	9 years	M
Obesity	Early-onset obesity	1 year	M
Puberty	Peripheral precocious puberty (congenital adrenal hyperplasia vs. Leydig cell tumor)	6 years	M
	Central precocious puberty	4 years	F
	Central precocious puberty	4 years	F
	Central precocious puberty	2 years 1 month	F
	Premature thelarche	1 year 6 months	F
	Premature thelarche	1 year 5 months	F
	Gynecomastia, rule out prolactinoma or tumor	13 years	M
	Gynecomastia secondary to HIV therapy (Effavirenz)	14 years	M
Thyroid	Graves' disease	14 years	NA
	Rule out hyperthyroidism	10 years	M
	Severe primary hypothyroidism	6 years	M
	Rule out congenital hypothyroidism	2 months 1 week	M
	Rule out congenital hypothyroidism	2 months	M
	Abnormal thyroid function tests	3 months	M
	Sick euthyroid	7 months	F
	Anticipated thyroidectomy due to cervical mass	3 months 2 weeks	F

### Consultation Evaluation

Detailed quality evaluations were available for 18 (38%) of cases.

#### Referring Physician's Quality Evaluation

All referring physicians who provided a qualitative response thought that their case was sent to an appropriate expert and that they received an answer sufficiently quickly. Sixteen (89%) thought the answer was well-adapted to their environment and 13 (72%) felt they were able to follow the advice given. Five (28%) felt they were not able to follow the advice and cited as reasons patient loss to follow-up (*n* = 2), no urgency to treat (*n* = 1), inability to find suggested treatment modality locally (*n* = 1), and no reason provided (*n* = 1). All but one referrer found the advice helpful, and out of the 18 cases that were qualitatively evaluated, the consultation helped to clarify the diagnosis in 16, assisted with management in 17%, improved patient's symptoms in 14, and improved function in 14.

##### Perceived benefits

Themes that emerged regarding benefits of the consultations were academic instruction, setting-adapted advice beyond the scope of guidelines or textbooks, and advancement in the diagnostic process. For example, one pediatrician from Myanmar commented: “*We can't do most of the investigation here, as we have to send [them to] Thailand, it's very expensive. […] I did just urine ketone level and serum insulin in a child with persistent hypoglycemia and high glucose infusion rate. We can't do other investigations. So, this consultation is very useful for me to have a diagnosis confidently.”* Expressed frustrations were loss to follow-up and lack of progress in finding a diagnosis.

Fifteen referrers thought the eventual outcome for the patient will be beneficial for the patient. When asked about educational benefit, 14 of 17 respondents thought the reply received was beneficial to their education. Of 18 respondents, only 9 thought that the consultation was cost-saving for the patients or families, and only 7 of 18 respondents thought the consultation was cost-saving for the hospital.

In the qualitative comments, the ability to access specialist care and to make a diagnosis, and parental reassurance surfaced as themes that referrers identified as offsetting any cost. Pediatricians in Haiti commented:“*…it benefits everyone: patients, parents, residents, staff, hospital and, in short: the country.”*; “*Because if we had not had the opinion of a specialist, in the context of our limitations, his case could have been misdiagnosed.”*

The perceived cost savings that surfaced in the comments were the ability to obtain specific exams, the context-adapted advice, and improved knowledge among health professionals: “*We were able to find a quick and accurate interpretation of the hand radiograph used to evaluate bone age. We have the opinion of pediatric endocrinologists, a specialty currently absent in Haiti.”*

Themes that emerged from the referring physician's additional comments on their experience with the consultation platform were gratitude for the help received, a perceived increase in knowledge, and the ability to make a diagnosis. Pediatricians from Haiti and Myanmar commented: “*The case was very interesting: it allowed me to increase my knowledge through the discussions I had with the specialist. Thank you so much.”;“It's very useful for both our children and me. It's my first experience in such a case […] so it's difficult to recheck the lipid level and CK [Creatine Kinase]. […] The dietary advice is very beneficial.”; “That's the first time I manage a case like that. It's very interesting, and I learned a lot.”; “I deeply appreciate the time spent on my case and the prompt response I received.”*

### Urgent Advice

Between August 2017 and April 2019, a total of 129 physicians registered onto the text messaging group and responded to questions concerning 13 cases (Table 5), exchanging 325 messages. Ten cases were deemed appropriate for urgent advice, while three were not. The most common reason for seeking advice were disorders of glucose metabolism (diabetes and hypoglycemia), and ambiguous genitalia. The median time to response by a consultant physician was 13.5 min (IQR 5, 56). Six of the thirteen cases were transferred to the store-and-forward consultation platform for further consultation and discussion.

**Table 5:** Reasons for referral and consultant diagnoses of urgent consultations by endocrine category.

**Endocrine Category**	**Reason for referral**	**Consultant diagnosis/recommendation**	**Age**	**Sex**
Adrenal	Ambiguous genitalia, salt-wasting	Adrenal crisis, salt-wasting Congenital adrenal hyperplasia	Infant	F
Diabetes	New onset diabetes with severe ketoacidosis	New onset diabetes with severe ketoacidosis	Child	Unknown
	New onset diabetes; severe ketoacidosis and cerebral edema	New onset diabetes; severe ketoacidosis and cerebral edema	Child	Unknown
	Hyperglycemia	Neonatal diabetes	2 days	Unknown
	Hyperglycemia, seizures	Neonatal diabetes	2 months	M
	Hyperglycemia	Neonatal diabetes vs. stress hyperglycemia, seizures, hypocalcemia	Infant	Unknown
Hypoglycemia	Hypoglycemia	Hypoglycemia likely due to hyperinsulinism, rule out Beckwith-Wiedemann Syndrome	Infant	Unknown
	Hypoglycemia	Hypoglycemia	7 years	M
Disorders of sexual development	Ambiguous genitalia	Hymenal skin tag	Infant	F
	Ambiguous genitalia	Ambiguous genitalia	Infant	Unknown
Puberty	Precocious puberty	Central precocious puberty	Child	F
	Breast asymmetry	Physiologic breast	Adolescent	F
	Gynecomastia, galactorrhea	Physiologic puberty	13 years	M

## Discussion

To our knowledge, this is the first study to demonstrate that a store-and-forward telemedicine network can facilitate access to high-quality specialized pediatric endocrine care in low- and middle-income countries for non-urgent disorders. The results of our evaluation show that close to fifty children with often complex endocrine disorders benefited from effective and timely endocrine consultations. Over 80 referring physicians from low-income countries who were predominantly based at tertiary care centers are now able to efficiently access the opinion and advice from a pediatric endocrinologist in four different languages. In the referring physician's perspective these remote consultations not only improve patient care most of the time, but also provide an important learning opportunity. Analysis of our text messaging network demonstrates that quick, more informal advice can be obtained in urgent situations.

### Global Pediatric Endocrine Teleconsultation

Reports on experience with teleconsultation for pediatric endocrine conditions are scarce and are unavailable from low- and middle-income countries. One Canadian study investigating access to an adult teleconsultation network for patients located in remote areas of Northern Canada showed that 7% of consults were for endocrinology, although details were not available (10). A telepediatric service in Queensland, Australia, reported 13% pediatric diabetes and 3% endocrine consultations, but the report focused on cost assessment and did not provide detail on the pediatric endocrine consultations (4). While there is a clear need and demand for better access to specialty pediatric endocrine consultation even in high-income settings (5), this is even more relevant to low- and middle-income countries who need advice from specialists familiar with the constraints in resources, diagnostics and therapeutics, and who can adapt their advice to the local setting, taking language, and cultural aspects into account. More studies are needed to evaluate the impact of teleconsultation on health care access, risks and benefits to patients and referring providers, quality of consultations, and cost-effectiveness in low-resource settings.

### User Profile and Platform Use

Most referring and consulting physicians on our network are affiliated with a tertiary care university hospital center. The percentage (89%) was almost double as compared to the 45% reported from the Médecins Sans Frontières (MSF) network on the same platform (11). This most likely reflects the subspecialized and academic nature of pediatric endocrinology but may also suggest a selective uptake of platform use by the physician population targeted. Further dissemination of our platform to primary care physicians and pediatricians in offices, clinics, and community hospitals could sway the proportion of consults received toward primary care-based referrals. Taking the higher uptake in Haiti through the PEEP-H activities as an example, our group has liaised with other international on-site teaching events including the ESPE-led schools in Eastern Europe (Winter School), North Africa (Maghreb School), and Central Asia (Caucasus and Central Asia School) so these can act as channels of communication for the recruitment of potential referrers.

Despite a clear need for subspecialty expertise in low-income settings, our teleconsultation services have remained under-utilized. Uptake in Haiti was likely higher due to promotion of the platform during onsite teaching activities by PEEP-H professors and the PEEP-H coordinator's dissemination activities. Of ~300 pediatricians who practice in Haiti 38 (13%) have registered. Most were repeatedly invited, had several opportunities to register, or were provided with in-person or remote help to register online. The registration process *per se* may be a barrier due to low comfort level with electronic device and online platform use. In contrast, only a small number of international referring physicians have registered, all of whom seemed to be highly motivated pediatricians who were proactively searching for a way to obtain input on their challenging case(s). Our global uptake through GPED may have been less rapid because promotion strategies have not reached our target population. In an attempt to improve our reach we assigned regional point-persons across five continents in 2019 who will work with their local and regional referral network and with pediatric professional organizations to improve platform visibility, access, and use. Work is ongoing to seek information on barriers and facilitators from referring physicians. Observations from two recent reports from Brazil suggest adequate training and encouragement of providers as well as integration of consultations into regular work hours are opportunities to improve teleconsultation use and dissemination (12, 13), which may well apply to our network as well.

Further, while registration to the consultation platform was high in Haiti, the number of cases referred is likely to be below the number of cases that may benefit from consultation. As in many low-income countries, access to specialty consultation in Haiti has previously been severely limited and the culture of seeking advice from a subspecialist has not been established as a routine. Anecdotally, a referral only felt justified to many pediatric providers in Haiti if their own attempts at diagnosis and treatment were unrevealing. They only proceeded with teleconsultation if referral information (history, physical exam, initial diagnostic work-up) was felt to be high-quality and worthy of sending to a specialist. Despite ease of platform use and despite its availability on mobile devices and full function even with slow internet connections (11), referrers may lack consistent computer, mobile phone or internet access, may not be comfortable typing, and may perceive the time it takes to send a consultation as a barrier. These factors may increase the threshold to seek remote advice.

### Quality Assessment and Cost

Our quality statistics overall reflected a low-frequency but high-quality and efficient use of our network. Our answer delay of 43.1 h largely resulted from a higher allocation delay and was longer than that reported in a previous study that used the Collegium Telemedicus platform (14), but similar to the 2-day response time reported from a Canadian network (1). However, most cases in pediatric endocrinology tend to be subacute or chronic, such that a delay of 48 h or less seems quite appropriate. In addition, we offered urgent case discussion via a separate urgent-response but less formal consultation forum where time to response was within less than an hour for all but three cases. Our response rate to detailed feedback requested from referrers after each case was 38%, which is higher than the 12% reported by the largest Collegium Telemedicus supported network and similar to their second largest network (42%) (8). The overall high satisfaction by referrers with impact on clinical care and education mimics evaluations from previous reports (11) but needs to be interpreted with some caution as there is a possibility these evaluations are biased by self-selection of referrers who were particularly satisfied with the consultation process. While we did not formally evaluate consultant experiences in this study, earlier reports and informal follow-up with our consultant specialists revealed insufficient follow-up on consultations (11). Whether the network manager or coordinator could encourage and enhance referrer-consultant interaction to facilitate shorter answer delays and ensure follow-up needs to be studied. Finally, the up to 50% cost savings reported in our network are closer to 20% in other networks (8). In both cases, however, cost-saving was not measured objectively but rather based on referrer's subjective impression. In addition, as all specialist services and platform use were provided free of charge, reimbursement of specialists, and platform fees were not taken into account. A thorough cost analysis from Australia demonstrated significant cost savings of their teleconsultation system above a certain number of referrals (4).

### Conclusion

In summary, pediatric endocrine remote store-and-forward consultations in low-resource settings result in a productive referrer-consultant communication enhancing and frequently replacing the need for a face-to-face visit. Referring physicians perceive a clinical and educational benefit from these interactions and see a potential for cost-saving. Despite this, global scale-up of network registration and use has been slow and merits investigation into barriers and opportunities for improvement. Further study is needed to formally evaluate the cost-effectiveness, long-term sustainability, and feasibility of integrating teleconsultation into the locally existing healthcare infrastructure.

## Data Availability Statement

The datasets generated for this study are available on request to the corresponding author.

## Ethics Statement

Ethical review and approval was not required for the study on human participants in accordance with the local legislation and institutional requirements. Written informed consent from the participants' legal guardian/next of kin was not required to participate in this study in accordance with the national legislation and the institutional requirements.

## Author Contributions

JO conceptualized the study, extracted and analyzed the data, and wrote the first draft of the manuscript. MC performed a literature review and revised the manuscript. RL contributed to data collection, analysis and interpretation, and revised the manuscript. The PEEP-H working group (FS, RE, RS GV, JC, RL) critically reviewed the manuscript drafts. All authors approved of the final version of the manuscript.

### Conflict of Interest

The authors declare that the research was conducted in the absence of any commercial or financial relationships that could be construed as a potential conflict of interest. The reviewer MD declared a past co-authorship with one of the authors GV to the handling editor.
